# A Study of Anatomical Variability in the Position of the Cystic Artery During Laparoscopic Visualization at Kempegowda Institute of Medical Sciences (KIMS) Hospital, Bengaluru

**DOI:** 10.7759/cureus.85159

**Published:** 2025-05-31

**Authors:** Sudhir M, Nitish S, Nikita Galani, Kailash J, Sachin BR

**Affiliations:** 1 Department of General Surgery, Kempegowda Institute of Medical Sciences, Bangalore, IND

**Keywords:** anatomical variability, calot’s triangle, cystic artery, cystic duct, laparoscopic cholecystectomy

## Abstract

Background: Laparoscopic cholecystectomy is the gold standard for gallbladder removal, requiring precise knowledge of Calot’s triangle to prevent complications. Variations in the cystic artery’s length, course, and position pose challenges in achieving a critical view of safety. Misidentification can lead to bile duct injury or bleeding, often necessitating conversion to open surgery. Careful dissection guided by anatomical understanding is essential for safer procedures.

Aim: This study aims to identify the prevalence of anatomical variations in the position of the cystic artery in relation to the cystic duct in Calot’s triangle among patients undergoing laparoscopic cholecystectomy at a tertiary care hospital.

Methods: A prospective observational study was conducted to identify intraoperative variations in the position of the cystic artery in relation to the cystic duct in patients undergoing laparoscopic cholecystectomy over 19 months. A total of 100 patients aged above 18 years, posted for cholecystectomy, were included. Patients with a history of previous abdominal surgeries and those cases converted to open surgeries were excluded. Positions of the cystic artery were categorized into posteromedial, posterolateral, anterior, and others.

Results: Out of 100 cases of laparoscopic cholecystectomy, 71 patients were females and 29 were males, showing a female predominance. Moreover, 87 patients had a cystic artery posteromedial to the cystic duct, seven had a cystic artery at posterolateral position to the cystic duct, five patients had anterior to the cystic duct, and in one patient, it was absent. In one patient, it couldn’t be made out due to necrosis. There was no incidence of any postoperative bile leak in any patients.

Conclusions: The most common position of the cystic artery in Calot’s triangle was posteromedial (87%) to the cystic duct. A thorough knowledge of the anatomy and variations of the extrahepatic biliary tract and arterial supply is essential for reducing bleeding, preserving visibility, and avoiding damage to other biliary and vascular structures. This understanding can guide us toward safer dissection of Calot’s triangle and conducting a successful laparoscopic cholecystectomy.

## Introduction

In India, the prevalence of cholelithiasis, commonly referred to as gallstone disease, varies and is increasing to 2%-29 % [1,2], whereas globally, it ranges from 10% to 20% [2,3]. In different communities across India, the prevalence of gallbladder stones varies significantly, with North Indians experiencing a two- to fourfold higher prevalence than South Indians [4]. Laparoscopic cholecystectomy has transformed the surgical approach to gallbladder removal, offering patients faster recovery, smaller incisions, and less postoperative pain compared to the traditional open method: A three- to four-day hospitalization has been transformed into an outpatient procedure, several weeks of recovery have been reduced to one week, and pain has significantly decreased. However, these benefits do not come without risk, most notably a doubling of the rate of major biliary tract injury and, in rare cases, vascular and intestinal injuries resulting from peritoneal access misadventures [5].

In addition to possessing adequate anatomical knowledge of related areas, understanding and recognizing Calot's triangle is crucial for successful laparoscopic cholecystectomy [6]. The cystic artery's anatomical variations have been examined in several previous studies. Calot described a triangular region formed by the cystic duct, right hepatic duct, and lower margin of the liver in 1891 [7]. Later, Rocko et al. outlined potential variations in Calot's triangle in 1981 [8]. Typically, the cystic artery originates from the right hepatic artery in 70%-80% of instances and travels within the cystohepatic triangle, positioned to the right of the common hepatic duct [9,10]. Iatrogenic injuries of the extrahepatic biliary tree and neighboring blood vessels are not rare [11]. Complications arising from bleeding in the cystic artery are particularly concerning during laparoscopic cholecystectomy, as they compromise visibility in the abdomen. The frequency of conversion to open surgery due to blood vessel injuries varies widely, reported between 1.2% and 6.62% [12,13]. Notably, bleeding in Calot’s triangle, including cystic artery injuries, has been associated with conversion rates of 11.8% to 17.6% [14].

Strasberg et al. [15] recommended a three-step surgical approach to prevent such iatrogenic damage to vascular and bile duct systems. Blind dissection of the Calot's triangle, including the hepatoduodenal ligament, is the first step. Since it deals with the blind dissection in the Calot's triangle, the first step in the critical view of safety is the most important part of the process. Because the relevant artery is not visible throughout this step, there is a danger of vascular injury, just like with any other blind surgery. Hence, surgeons who are not yet familiar with the handling of an anatomically abnormal cystic blood supply need to be more aware of the precise anatomy of the extrahepatic biliary tree [6].

Additional research on the cystic artery’s anatomical variations, specifically in the Calot’s triangle, and how the different cystic artery positions affect intraoperative decisions and patient outcomes needs to be carried out among different populations. Hence, this study was carried out to identify the prevalence of anatomical variations in the position of the cystic artery in relation to the cystic duct in Calot’s triangle among patients undergoing laparoscopic cholecystectomy at a tertiary care hospital, the Kempegowda Institute of Medical Sciences and Research Center in Bengaluru, South India.

## Materials and methods

Following ethical clearance from the Institutional Review Board, a prospective observational study was conducted on 100 patients aged 18 years and above, who provided informed consent, met the eligibility criteria, and were scheduled for laparoscopic cholecystectomy during the study period from March 2021 to October 2022. All patients aged 18 years and above, undergoing laparoscopic cholecystectomy, were included in the study. Patients with a history of prior abdominal surgeries and patients who were converted to open surgery from laparoscopy, due to nonvisualization of the Calot’s triangle, were excluded. All patients underwent detailed clinical examinations, laboratory investigations, and radiological imaging. Laparoscopic cholecystectomy was performed in all the patients. A standard four-port procedure was used to proceed with cholecystectomy. The technique did not change during the course of the study. Briefly, a Veress needle was inserted in the periumbilical region, and initial peritoneal access was achieved. CO_2 _was insufflated, and pneumoperitoneum was achieved. Three additional ports were inserted: one subxiphoid port (10 mm) and two subcostal ports (5 mm). After adequate dissection, the cystic artery and duct were identified. Photographs were taken intraoperatively to determine the position of the cystic duct in relation to the cystic artery, and this was recorded on a proforma. The positions of the cystic artery were categorized on the basis of the position of the cystic artery in relation to the cystic duct in the Calot's triangle as posteromedial, posterolateral, anterior, and others.

## Results

**Figure 1:**
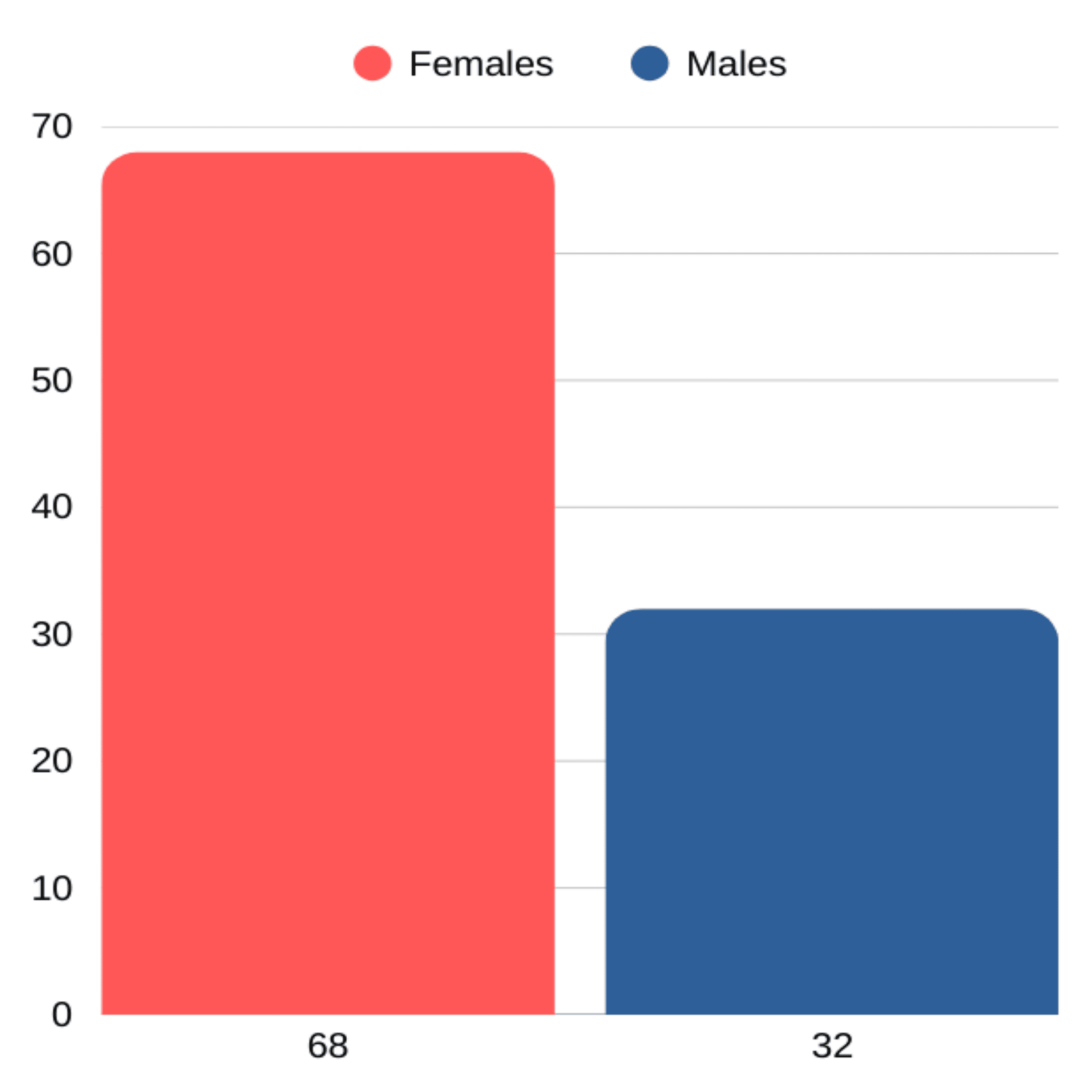
Distribution of study participants by gender

**Figure 2:**
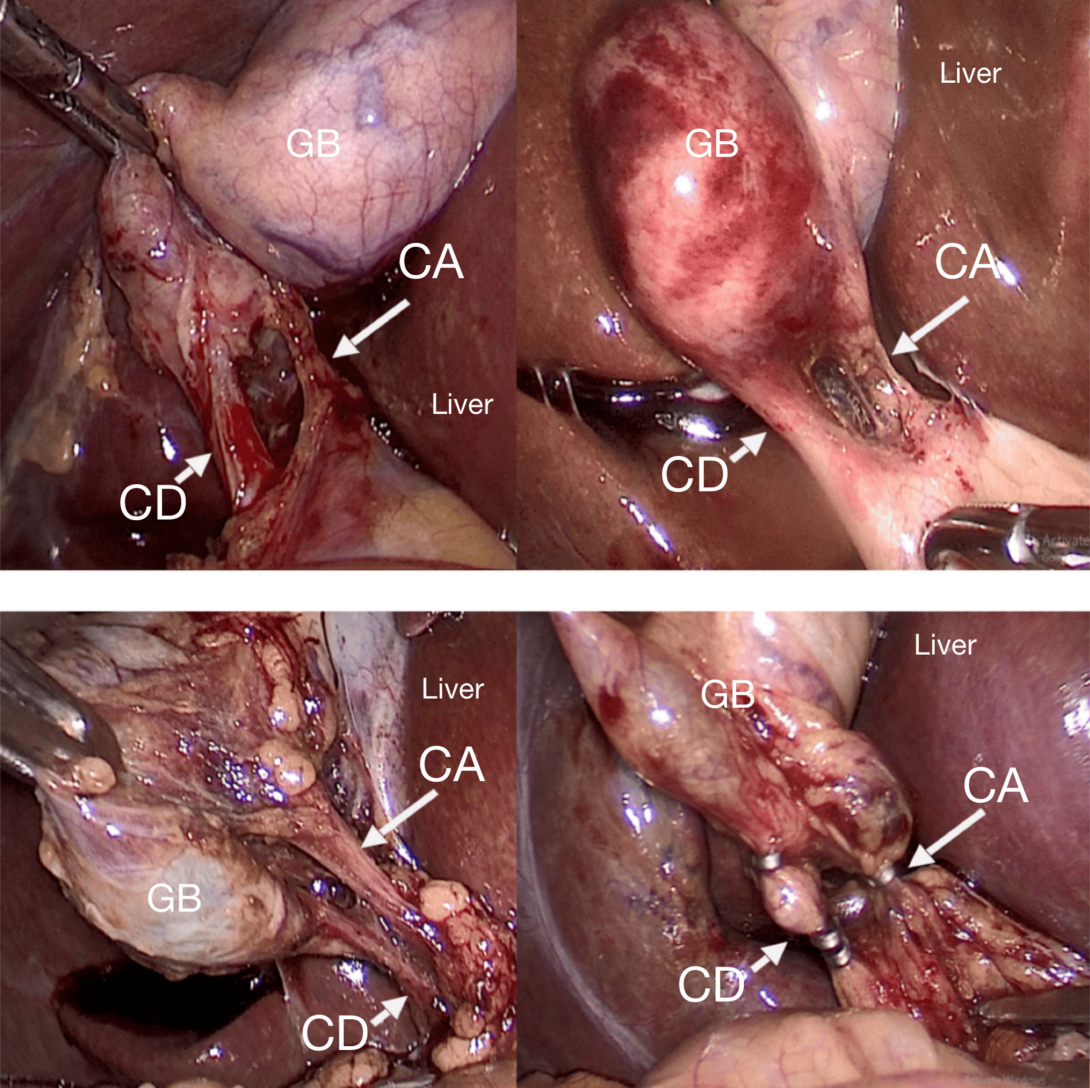
Cystic artery is posteromedial to cystic duct CD: cystic duct; CA: cystic artery; GB: gall bladder

**Figure 3:**
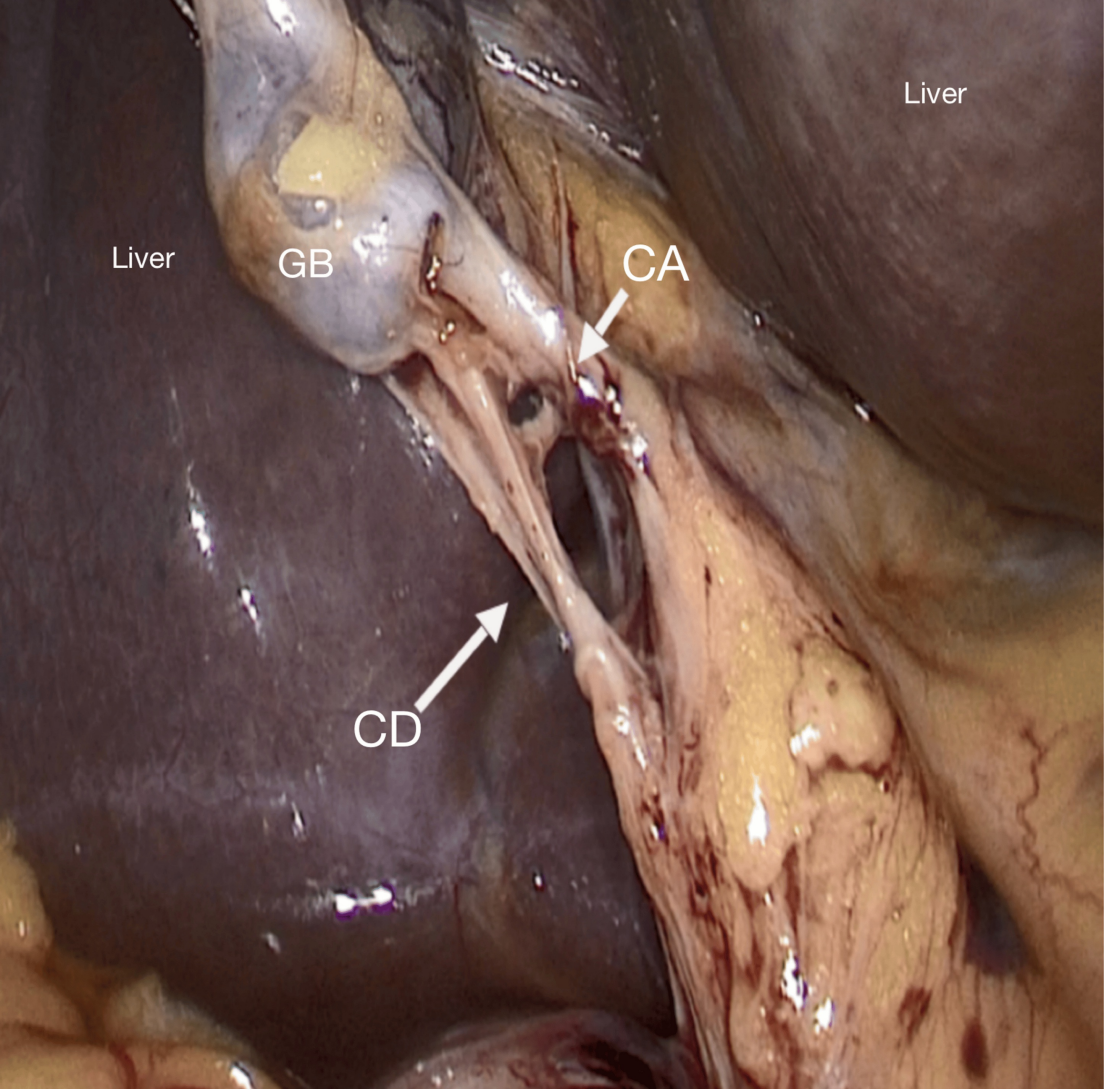
Cystic artery is posterolateral to the cystic duct CD: cystic duct; CA: cystic artery; GB: gall bladder

**Figure 4:**
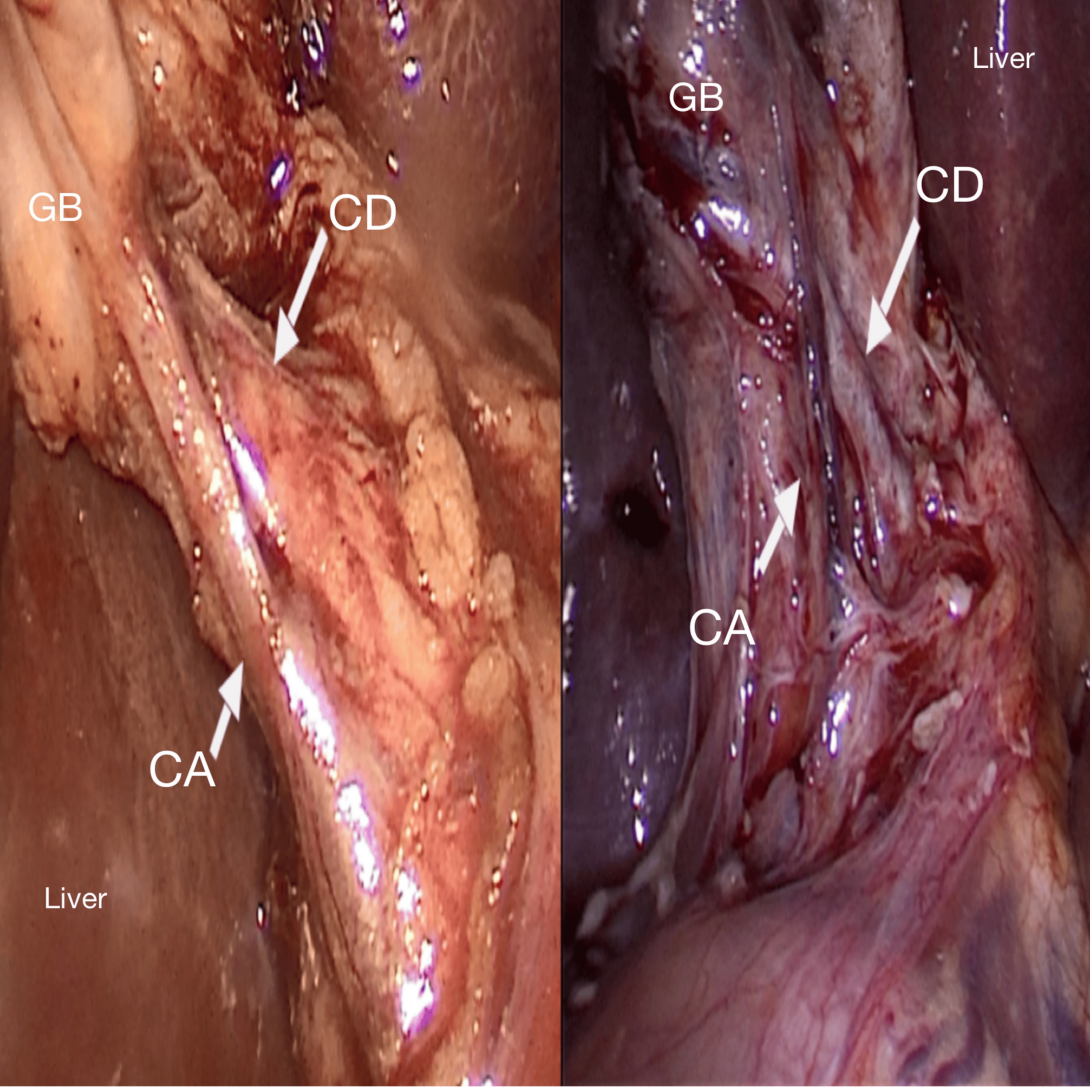
Cystic artery is anterior to the cystic duct CD: cystic duct; CA: cystic artery; GB: gall bladder

**Figure 5:**
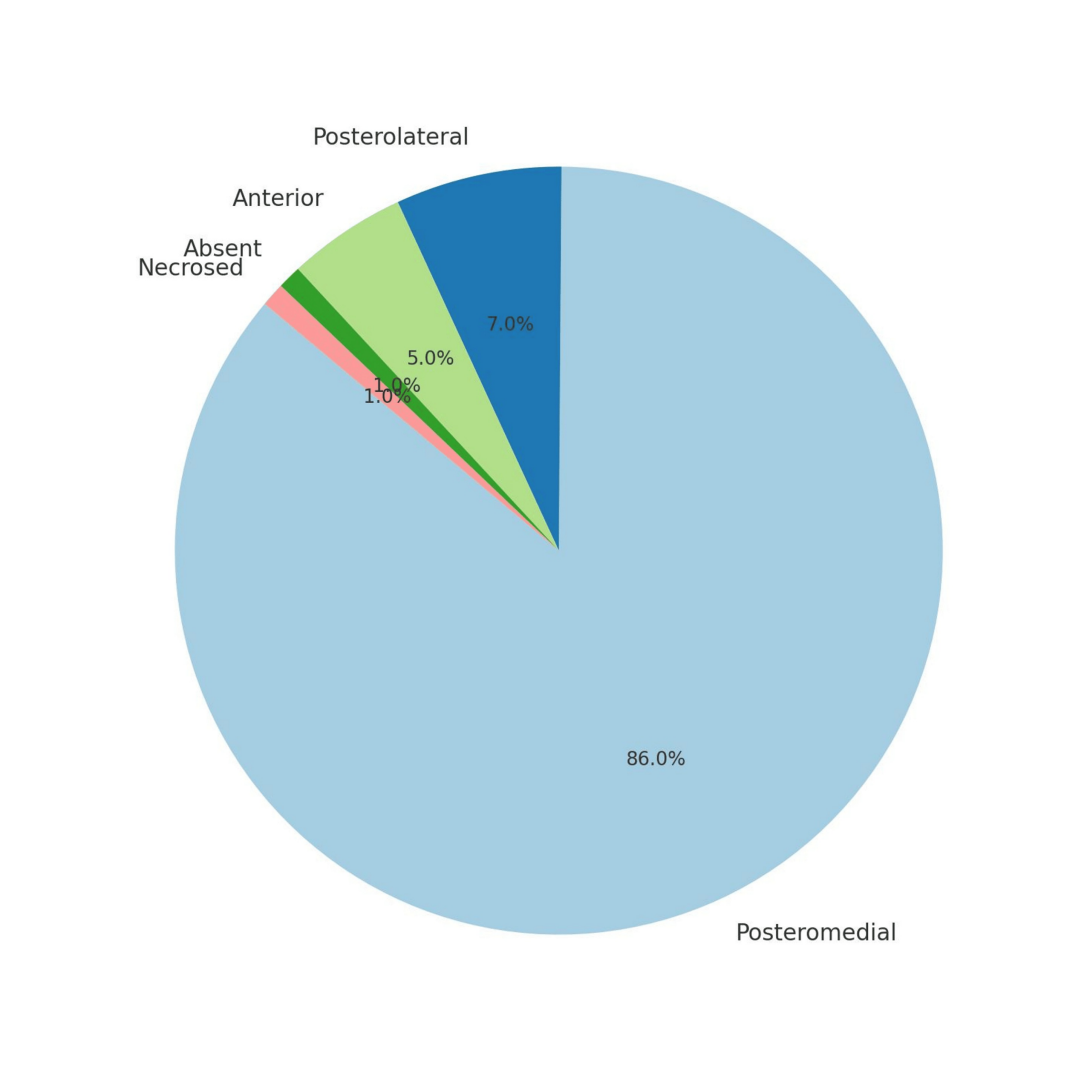
Distribution of the number of cases of anatomical variations of the cystic artery

Among the 100 participants, the largest age group affected was 41-50 years, accounting for 32% of cases (Table 1). Additionally, gallstones were more prevalent in females, with 68 women compared to 32 men, highlighting a notable female predominance in the condition (Figure 1). In the 100 laparoscopic cholecystectomy cases, the cystic artery was most commonly located posteromedially (Figure 2) in 86 patients, followed by posterolaterally (Figure 3) (7 patients) and anterior to the cystic duct (Figure 4) in five patients. In rare instances, the artery was absent/not found (1 patient) or necrosed (1 patient) (Figure 5). The age distribution of the subjects in the study is presented in Table 1.

**Table 1:** Age distribution of the patients in the study

Age groups (years)	Number	Percentage (%)
20-30	13	13
31-40	25	25
41-50	32	32
51-60	20	20
>60	10	10

## Discussion

Knowledge of the cystic artery and its variations is essential to perform safe cholecystectomies. Variations in position, size, and relationship with adjacent structures are common.

Out of the 100 patients, 68% were females. The results suggest that gender-specific factors play a crucial role in the development of cholelithiasis. The higher prevalence of gallstones in women can be attributed to hormonal influences, such as estrogen, which increases cholesterol saturation in bile and decreases gallbladder motility. Additionally, factors such as pregnancy and the use of oral contraceptives further elevate the risk of cholelithiasis in women. These findings are consistent with previous studies [6,16,17].

The maximum number of patients was in their forties. The frequency of gallstones increases with age, escalating markedly after age 40 to become four to 10 times more likely in older individuals [17,18]. Gallstone disease is the most common biliary tree disease affecting middle-aged females in their reproductive age group.

In the 100 laparoscopic cholecystectomy cases, the cystic artery was most commonly located posteromedially (86 patients), which is considered to be normal anatomy. A review [19] of the different forms of the cystic artery documented through the analysis of cholecystectomies indicated that in the majority of the included studies (54.5%), the typical anatomical pattern of the cystic artery was the most often occurring type. This is the typical trend for the majority of people. The cystic artery originates from the right hepatic artery and travels through Calot's triangle to the right and posterior to the common hepatic duct and then passes superiorly to the cystic duct at the gallbladder's neck, bifurcating into a superficial and deep branch to supply the gallbladder and the cystic duct [20].

In the present study, in seven patients, the cystic artery was posterolateral to the cystic duct, similar to that reported from West Bengal (7.7%) [21]. Among the 1850 patients operated for laparoscopic cholecystectomy, Fateh et al. reported that the second most common position was found to be the cystic artery posterolateral to the cystic duct in 10 (5.208%) patients [6].

In this study, the cystic artery was found to pass anteriorly to the cystic duct in five patients, a finding consistent with previous reports. Balija et al. [22] documented a 4.5% prevalence, aligning with the current observations. A recent study from West Bengal by Sengupta et al. [21] noted the anterior position to be the least common (2.5%). In contrast, another Indian study by Gupta et al. [23] recorded a higher frequency (10.7%), identifying this position in 32 patients. A review of 22 studies revealed that in 36.4% of cases, the cystic artery was positioned anteriorly to the common hepatic duct or cystic duct, reinforcing the importance of careful surgical technique in this region [19].

The absence or necrosis of the cystic artery is an uncommon but important anatomical variation that can complicate laparoscopic cholecystectomy. In this study, one patient had a missing cystic artery, while another had a necrosed artery. Fateh et al. [6] also documented that the cystic artery was absent in three (1.56%) patients. Similarly, Suzuki et al. [11] reported that 11.1% of cases lacked a cystic artery in Calot’s triangle, highlighting the variability of gallbladder vascularization. Additionally, Andall et al. [24] conducted a systematic meta-analysis of 9,800 cases, revealing that the cystic artery was absent in 0.34% of cases. While this review did not focus specifically on laparoscopic patients or define positional variations in laparoscopic views, it underscores the necessity for surgeons to recognize atypical vascular structures during procedures. Necrosis of the cystic artery may occur in the context of severe acute cholecystitis, where inflammation leads to vascular compromise. However, such cases are rare and usually present as part of a broader spectrum of severe gallbladder pathology (e.g., gangrenous cholecystitis), rather than as an isolated finding during routine laparoscopic cholecystectomy. These findings emphasize the importance of preoperative imaging and meticulous intraoperative dissection to mitigate surgical risks related to vascular anomalies.

The study on cystic artery positioning during laparoscopic cholecystectomy has several limitations. The sample size of 100 cases may be insufficient for representing all anatomical variations, and the research conducted at a single institution may not generalize to other settings with different patient demographics. Observer variability in identifying and classifying the cystic artery’s position could introduce bias, and the lack of specified preoperative imaging might limit the ability to anticipate variations. Additionally, the study lacks long-term follow-up data on surgical outcomes and complications, and there may be limited information on the management of cases where the cystic artery was absent or necrosed. Addressing these limitations in future research could enhance the understanding of anatomical variations and their impact on surgical outcomes.

## Conclusions

The study on cystic artery positioning during laparoscopic cholecystectomy underscores the prevalence of various anatomical configurations and their implications for surgical practice. The predominant finding of the cystic artery in the posteromedial position aligns with existing literature, highlighting its commonality and significance in surgical planning. The posterolateral and anterior positions, while less frequent, also reveal important variations that necessitate careful intraoperative management. Variability in prevalence across different studies emphasizes the need for flexibility and adaptability in surgical approaches.
